# Dune Blowouts as Microbial Hotspots and the Changes of Overall Microbial Activity and Photosynthetic Biomass Along with Succession of Biological Soil Crusts

**DOI:** 10.1007/s00248-023-02333-4

**Published:** 2023-12-29

**Authors:** Karolina Chowaniec, Jakub Styburski, Szymon Kozioł, Zofia Pisańska, Kaja Skubała

**Affiliations:** 1 Institute of Botany, Faculty of Biology, Jagiellonian University, Gronostajowa 3, 30-387 Kraków, Poland; 2 Doctoral School of Exact and Natural Sciences, Jagiellonian University in Kraków, Prof. S. Łojasiewicza 11, 30-348 Kraków, Poland

**Keywords:** Biological soil crust, Dehydrogenase activity, Chlorophyll, Inland dunes, Succession

## Abstract

Biological soil crust (BSC) constitutes a consortium of cyanobacteria, algae, lichen, mosses, and heterotrophic microorganisms, forming a miniature ecosystem within the uppermost soil layer. The biomass of different organisms forming BSC and their activity changes along with succession. Previous studies focused primarily on BSC in hyper-arid/arid regions, whereas the ecophysiology of BSC in temperate climates is still not well recognized. In order to determine changes in overall microbial activity and photosynthetic biomass in BSC at different stages of the succession of inland sandy grasslands, we analyzed dehydrogenase activity and determined the content of photosynthetic pigments. We also compared these parameters between BSC developed on the dune ridges and aeolian blowouts in the initial stage of succession. Our study revealed a significant increase in both photosynthetic biomass and overall microbial activity in BSC as the succession of inland shifting sands progresses. We found that chl *a* concentration in BSC could be considered a useful quantitative indicator of both the presence of photoautotrophs and the degree of soil crust development in warm-summer humid continental climates. The photosynthetic biomass was closely related to increased microbial activity in BSC, which suggests that photoautotrophs constitute a major BSC component. Dune blowouts constitute environmental niches facilitating the development of BSC, compared to dune ridges. High biomass of microorganisms in the dune blowouts may be associated with a high amount of organic material and more favorable moisture conditions. We conclude that deflation fields are key places for keeping a mosaic of habitats in the area of shifting sands and can be a reservoir of microorganisms supporting further settlement of dune slopes by BSC.

**Supplementary Information:**

The online version contains supplementary material available at 10.1007/s00248-023-02333-4.

## Introduction

Biological soil crust (BSC) was described as the “living skin” on the soil surface that occurs in many water-limited ecosystems around the world [1]. In arid and semi-arid areas, vegetation cover is often sparse or absent; however, in open spaces between vascular plants, the soil surface is not bare of life, but covered by a community of highly specialized organisms. BSC constitutes a consortium of photoautotrophic cyanobacteria, algae, lichens, and bryophytes growing together with heterotrophic fungi, bacteria, and archaea, forming a miniature ecosystem occurring within the uppermost layer of the soil [2]. They are considered pivotal ecological elements since, at a microsite scale, BSC enables the development of plants that might otherwise be severely constrained by harsh abiotic conditions. BSC plays an important role in the C and N cycle, contributes to soil fertility, prevents erosion, improves soil microbial diversity, and facilitates vascular plant colonization [3]. Interest in understanding the role of biocrusts as ecosystem engineers in arid areas has increased considerably during the past two decades. Various types of organisms that create BSC share some interesting physiological traits. They are all capable of drying out and temporarily suspending respiration without negative effects [4]. This property allows them to survive in extremely adverse environmental conditions. Most of them equilibrate their water content with atmospheric humidity or soil surface moisture content. Autotrophic poikilohydric organisms generally become photosynthetically active very quickly, producing carbohydrates minutes after getting wet [5].

Succession is related to predictable changes in species composition over time as an ecosystem develops or recovers from a disturbance. In initial dryland ecosystems, cyanobacteria, algae, fungi, mosses, and lichens are the first organisms to colonize the substrate and form BSC [6]. In the successional pathway of BSC, the pioneers are cyanobacteria, then green algae, while lichens and mosses emerge later [7, 8]. Cyanobacteria crusts are formed quickly due to their special characteristics, including relatively rapid growth, migration, and their exceptional ability to survive drought, extreme temperatures, and pH. They can effectively accelerate C and N cycles in BSCs by increasing the biomass inputs and fixing N in soils, which contribute significantly to the formation of soil aggregates and the primary accumulation of C and N in soil [9]. Furthermore, they excrete gelatinous exopolysaccharides that influence the substrate stability and reduce soil substrate erosion [10]. Initial crusts composed of algae and cyanobacteria can also facilitate succession to later stages due to their ability to improve the surface microhabitat [11]. Subsequent colonization of lichens and mosses is facilitated by a gradual improvement of soil conditions, such as nutriment contents and physicochemical properties [12]. Lichens and mosses found in later stages of BSC succession are characterized by greater photosynthetic efficiency and carbon productivity than those of initial crusts. Many free-living fungi also interact with complex microbial communities, and such developed BSCs in dryland ecosystems have complex biodiversity, multifunctionality, and stability [13]. A recent study described the ongoing succession of BSCs, providing evidence of changes in the composition, diversity, and functioning of the BSC community [14]. Along with the succession of BSCs, their ecological functions vary, which is associated with the biomass of different organisms and their activity that differs between different successional stages (e.g., [15]). For example, both bacteria and fungi play important roles in the C and N cycle; however, signal intensities of different functional genes involved in C and N metabolism change significantly with BSC development with bacterial and fungal communities contributing differentially to C and N cycles during BSC succession [16]. Although BSCs of different ages exhibited the same dominant phyla, the proportions between them change with BSC development, and thus, the greater range of functional characteristics of bacterial communities compared to fungal ones in the initial stages of succession could be associated with higher diversity and richness of bacterial communities [16, 17].

Previous studies have focused primarily on BSCs in hyper-arid and arid regions with very low precipitation and very high temperatures, whereas the development, ecophysiological traits, and functioning of BSCs in temperate climates are still not well understood [18]. Moreover, investigations in temperate regions have mainly focused on floristic, taxonomic, and phytosociological, rather than functional aspects of BSC. BSCs are usually composed of multiple unrelated organisms that occur together on the soil surface. One organism studied out of several BSC organisms does not represent the ecological response of a complete BSC [19]. Thus, in the present study, we took a holistic approach to the BSC as a functional unit composed of a range of different organisms to identify the physiological traits of BSCs as a whole unit. This is crucial for a better understanding of the function and importance of BSC in initial habitats of temperate climates.

There are two key parameters that indicate the physiological status of the BSC. The first is the chlorophyll content, which determines the total biomass of autotrophic organisms and constitutes an excellent photosynthetic biomass indicator [5]. The second is dehydrogenase activity that readily indicates overall microbial activity in BSC and corresponds to the abundance and metabolic activity of both autotrophic and heterotrophic microorganisms [20]. Since physiological studies on BSC are rare, so far, only a few studies have been carried out on changes in the content of chlorophyll in BSC depending on the stage of succession (e.g., [8, 21]). Yeager et al. [21] found higher chl *a* concentration in BSC from the late succession stage in the Colorado Plateau, while the study on BSC from inland sand ecosystems in Germany showed no significant differences between initial and stable BSC [8]. Even less is known about the dehydrogenase activity (DHA) in BSC. Miralles et al. [22] analyzed DHA from different depths (crusts, middle, deep) and showed a decrease in DHA from the crust to the deep soil layer. Moreover, a comparison of DHA between lichen-dominated BSC and soil underlying them revealed higher DHA in BSC samples [23]. Finally, it was found that BSC significantly increased soil DHA, as revealed by Liu et al. [24] in revegetated areas of the Tengger Desert.

The aim of the study was to determine changes in overall microbial activity and photosynthetic biomass in biological soil crusts at different stages of the succession of inland sandy grasslands. We also aimed to compare these parameters between BSC developed on the dune ridges and dune blowouts in the initial stage of succession. We set the following hypotheses: (1) The biomass of photosynthetic organisms and overall microbial activity increases with succession. (2) The dune blowouts (deflation basins) are characterized by a greater abundance of microorganisms and biomass of photoautotrophs compared to the dune ridges. (3) Since cyanobacteria only comprise chlorophyll *a* and have the greatest share in the initial stages of succession, the chl *a*/*b* ratio would be clearly higher in the initial stages of sand colonization.

## Materials and Methods

### Study Area

**Fig. 1:**
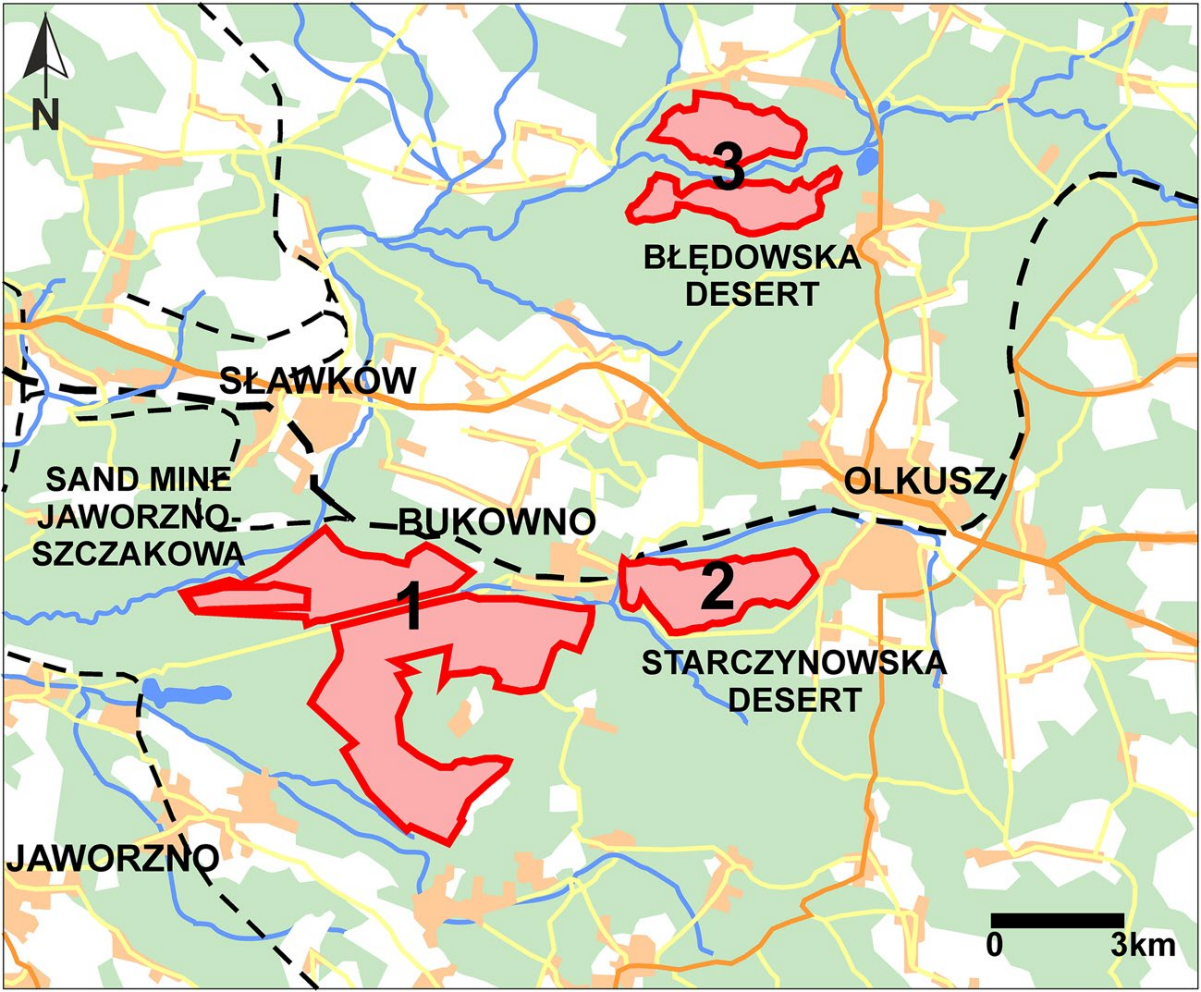
Location of the study sites in the Silesia-Cracow upland (S Poland)

The study covers one of the largest in Europe areas of airborne sands of approximately 150 km^2^, which are separated by forests or fields. This study includes three sites located in S Poland: (1) areas of the former Szczakowa sand mine in Bukowno, (2) Starczynowska Desert, and (3) Błędowska Desert (Fig. 1). The study sites have the same origin and are made up of quaternary fluvioglacial sands [25]. Before the twelfth century, the study area was covered by dense forests. Later, the forests were cut down for industrial purposes, and the sandy substratum was uncovered. This caused the activation of aeolian processes [26, 27]. These sites do not represent real climatic deserts, but during several hundred years of their existence, they were characterized by a specific “desert” landscape. Their existence is conditioned by natural factors, huge masses of sandy formations, and by anthropogenic factors, consisting in the continuous destruction of the forest cover [28]. In the absence of human activity, the deserts decreased in size, but due to secondary human intervention in some places, the process of succession started again from the beginning. In the following years, aeolian processes were restarted at the studied sites at different times and with varying intensity. At the first study site since the 1950s, sand began to be exploited with an area of approximately 32 km^2^, and these open-pit excavations of the Szczakowa sand mine are the largest mining excavations of this type in Poland and one of the largest in Europe [29]. The area of 29.64 km^2^ was reclaimed by planting trees in 1960–2005 [30]. In some places, mining work was finished, and the area was left without reclamation, where spontaneous succession takes place. The activation of aeolian processes took place along with the formation of small accumulation and deflation relief forms in the bottoms and slopes of the sand pit in areas not subjected to reclamation interventions [31]. At Starczynowska and Błędowska Deserts, the aeolian factor caused the formation of various accumulation and deflation forms, which were remodeled and transformed many times. In the landscape of these sites, the most distinct forms are dunes characterized by uneven distribution, different sizes and shapes. The deflation forms include blowouts and deflation troughs. The deflation blowouts constitute shallow (1,5–3 m deep) depressions of oval shapes [26, 27]. In 2014, a desert landscape was restored in the Błędowska Desert (under the Life + conservation program: LIFE09 NAT/PL/000259) in order to protect the complex of sandy natural habitats: thermophilic grasslands and inland dunes. Uncovering large sandy areas resulted in the activation of volatile sands. On the exposed surface of loose sands, the activation of aeolian processes leads to the increase of the currently existing deflation area.

**Fig. 2:**
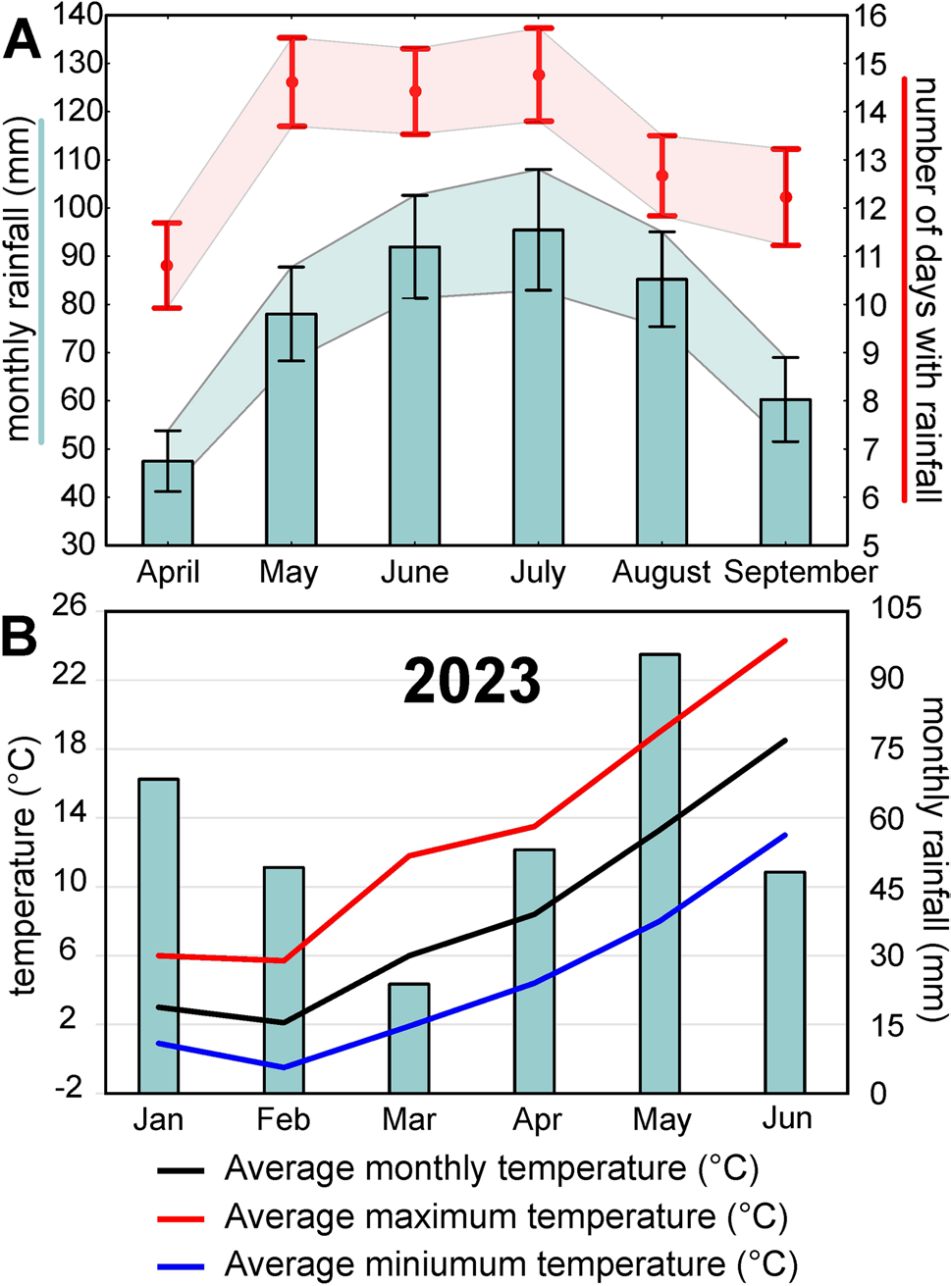
Climatic parameters of the study area. (**A**) The diagram presenting mean values and the 95% confidence interval for monthly rainfall and the number of days with rainfall in the spring–summer season. Based on averaged values for 1951–2022. (**B**) The diagram presenting temperature and monthly rainfall for the period of 6 months (in 2023) before collecting BSC samples. The raw data were obtained from the Institute of Meteorology and Water Management, National Research Institute (IMGW meteorological station – IMGW code: 250190390)

In the study area, W and SW wind directions are the dominant, and the proportion of coarse grains increases from the west to east, according to the prevailing wind direction [32]. Sands are lithologically uniform, consisting of rounded and well-rounded quartz grains, with the addition of feldspar, muscovite, garnet, zircon, tourmaline, chlorite, and glauconite [33, 34]. As regards soil characteristics, three soil development phases could be distinguished based on morphological features and accumulation of organic matter in soil: initial (regosols), transitional (arenosols and half-mature – podzolic soils in the initial stage of development), and mature (proper podzolic soils) [35]. The sand fraction clearly dominates in the soil, with a silt fraction up to several percent and a clay fraction up to 5%. The soil pH at each stage of succession is acidic and very acidic and ranges from 3.4 to 6.0 [35, 36]. As a rule, organic carbon and total nitrogen content in the soil increase with succession; however, it shows high differentiation depending on organic-humus horizon thickness [36, 37]. According to the Köppen–Geiger climate classification [38], the study area is located within a warm-summer humid continental climate (Dfb). Based on the data obtained from the nearest meteorological station, the mean annual temperature is 8.9 °C, and the mean annual precipitation sum amounts to 688 mm (average values calculated for 1951–2022, IMGW meteorological station – IMGW code: 250190390, data obtained from the Institute of Meteorology and Water Management, National Research Institute; see also Fig. 2A for rainfall conditions during the spring–summer season and Fig. 2B for temperature and monthly rainfall for the period of 6 months before sampling). The details on other climatic parameters in the study area are provided in Supplementary Table S1.

### Succession Stages

The natural series of the primary succession in the study area starts with BSC dominated by cyanobacteria and algae, followed by lichens, mosses, and grasses of the Koelerio glaucae-Corynephoretea canescentis class, especially from unions of Corynephorion canescentis and Koelerion glaucae. Next, single species of trees and shrubs gradually overgrow the area. At the optimum stage, pine forests (*Leucobryo-Pinetum*/*Cladonio-Pinetum*) are formed in which most of the psammophilous species retreat and are replaced by coniferous forest species [28, 35].

**Fig. 3:**
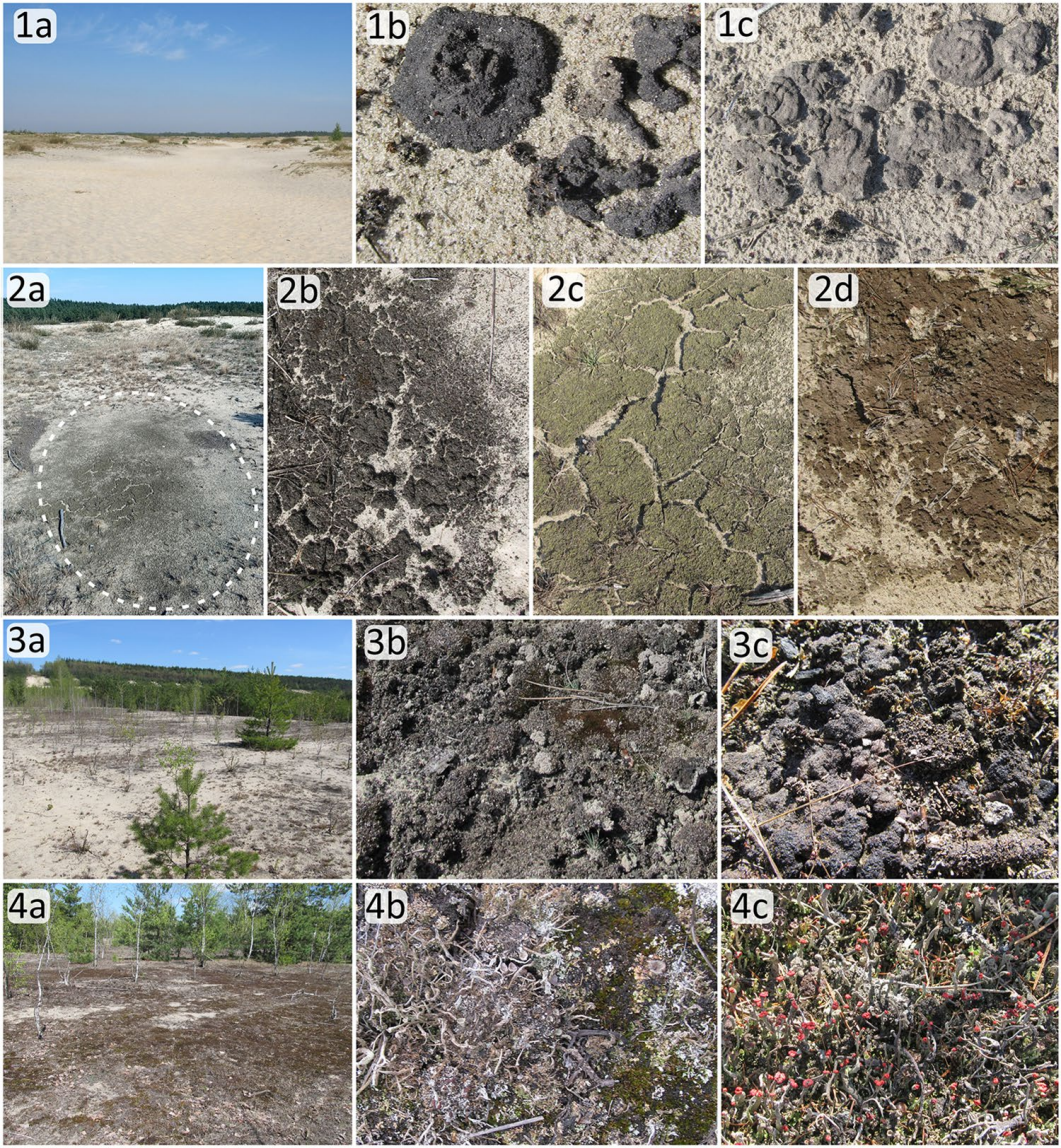
Exemplary photo presenting three succession stages and biological soil crusts (BSC). (1) initial stage, dune ridges (**a**), BSC (**b**, c); (2) initial stage, dune blowout (**a**), BSC (**b**–**d**); (3) middle stage (**a**), BSC (**b**, **c**); and (4) late stage (**a**), BSC (**b**, **c**)

Three stages of succession were defined for the purpose of the present study; all of them are present in the study area, which was confirmed in the preliminary research. The time of colonization initiation (beginning of the succession process) at particular sampling sites was determined on the basis of available databases, and in the absence of the data, it was determined on the basis of historical satellite images (geoportal.gov.pl). The succession duration range was within 5–30 years. Based on our field research and data from literature [35], we distinguished 3 succession phases to be analyzed (Fig. 3): (1) initial (up to 10 years): bare sand on the surfaces of the deflation fields and the accumulated aeolian sediments inhabited primarily by soil algae and cyanobacteria; (2) middle (10–20 years): colonization of cryptogamic species, mainly lichens (*Cladonia* spp. *Diploschistes muscorum*, *Stereocaulon incrustatum*) and mosses such as *Polytrichum piliferum* and *Ceratodon purpureus* and the first occurrence of vascular species (mainly grass *Corynephorus canescens*); and (3) late (20–25/30 years): *Spergulo morisonii-Corynephoretum canescentis* community with significant number of lichen and moss species and other species typical for this community.

### Sampling

The sampling was done in the summer season of 2023. Seventeen sampling sites of 2 m × 2 m size were established in the middle and late succession stages. Thirty-four sampling sites were designated in the initial stage of succession; 17 of them were located in the top part of dunes (hereafter referred to as dune ridges), and 17 were in the deflation fields (hereafter referred to as dune blowouts). At each sampling site, 10 BSC samples were randomly collected with a metal ring of 14 mm diameter (surface size: 153.9 mm^2^) that was used to obtain BSC samples for further analyses: 5 of them were designated for determination of chlorophyll concentrations, and 5 of them for dehydrogenase activity assessment. In this way, dune ridges (initial stage), dune blowouts (initial stage), and middle and late succession stages were represented by 85 BSC samples. The ring was pressed into BSC, and a sample within the ring was recovered. To prevent contamination, the ring was cleaned from particles and sterilized with 70% ethanol before and after each sample collection; sterile gloves were used during sampling. The BSC samples were packed into paper bags and transported to the laboratory. Then, each sample was freed of loose soil grains by tapping with a soft brush.

### Dehydrogenase Activity Assessment

Dehydrogenase activity (hereafter referred to as DHA) was determined using the method described by [39] with minor modifications. Fresh, homogenized BSC samples were placed in test tubes and mixed with 2 ml 3% TTC (Sigma-Aldrich) in 50 mM sodium phosphate buffer adjusted to pH 6.8. Then, the tubes were covered with aluminum foil and incubated in the dark for 24 h at 25 °C. After incubation, the samples were vortexed and centrifuged (MIKRO 200, Hettich, Germany) for 10 min at 3000 × *g*. The supernatant liquid was discarded. The TPF formed was extracted with methanol; 2 ml of methanol was added to each of the tubes and incubated for 30 min at 60 °C in a water bath (JWE 357, Elpin-Plus, Poland). The operation was repeated, and a total of 4 ml of methanol was used for extraction. Then, the samples were centrifuged, and the absorbance of the supernatant liquid was measured at 485 nm with a UV–Vis spectrophotometer (Shimadzu UV-1900i, Shimadzu Corporation, Japan). TPF concentration was calculated using a calibration curve (prepared according to the standard method).

### Determination of Chlorophyll Concentrations

Prior to chlorophyll extraction, the dry weight of each BSC sample was determined. Then, the samples were slightly sprinkled with distilled water to activate BSC organisms, which facilitated the subsequent chlorophyll extraction [40]. The samples were placed in glass test tubes covered with aluminum foil. Next, 3 ml of DMSO (≥ 99.9%, Sigma-Aldrich) with the addition of CaCO_3_ (≥ 99.0%, Sigma-Aldrich) was added to the samples. All tubes were placed in a water bath (JWE 357, Elpin-Plus, Poland) at 65 °C for 90 min. After the first extraction cycle, each sample was vortexed for 30 s, and 3 ml of fresh DMSO was added to the samples for the second extraction cycle. After the second extraction in the water bath for 90 min, the samples were centrifuged (MIKRO 200, Hettich, Germany) for 10 min at 3000 × *g*, and the absorption was measured with a UV–Vis spectrophotometer (Shimadzu UV-1900i, Shimadzu Corporation, Japan) at 648 nm, 665 nm, and 700 nm. If absorption values at 665 nm were above 1.0, the sample was diluted 1:1 with DMSO, and the equation was adjusted accordingly. During the chlorophyll extraction procedure, the samples were kept in semi-dark conditions to prevent the degradation of chlorophyll. The chlorophyll concentrations were calculated according to [40]

Total amount of chl *a* + *b* in a sample:

$$\mathrm{Chl }a+b \left(\mu g\right)=\left[\left({A}_{665}-{A}_{700}\right)\times 8.02+\left({A}_{648}-{A}_{700}\right)\times 20.2\right]\times DF\times S$$

Chl *a* + *b* concentration on surface area:

$$\mathrm{Chl }a + b \left(mg\times {m}^{-2}\right)=\frac{\mathrm{Chl }a+b \left(\mu g\right) }{\mathrm{AR }\times 1000}$$

Chl *a* + *b* concentration on dry weight:

$$\mathrm{Chl }a + b \left(\mu g\times {g}^{-1}\right)=\frac{\mathrm{Chl }a+b \left(\mu g\right) }{DW}$$

Total chl *a* amount *n* in sample:

$$\mathrm{Chl }a \left(\mu g\right)=\left[\left({A}_{665}-{A}_{700}\right)\times 12.19\right]\times DF\times S$$

Chl *a* concentration based on surface area:

$$\mathrm{Chl }a \left(mg\times {m}^{-2}\right)=\frac{\mathrm{Chl }a \left(\mu g\right) }{\mathrm{AR }\times 1000}$$

Chl *a* concentration based on dry weight:

$$\mathrm{Chl }a \left(\mu g\times {g}^{-1}\right)=\frac{\mathrm{Chl }a \left(\mu g\right) }{DW}$$

where:

*A*_*x*_ is the absorbance at a certain wavelength, *DF* is the dilution factor, *S* is the amount of DMSO (ml), *AR* is the area (m^−2^), and *DW* is the dry weight (g).

### Statistical Analysis

One-way analysis of variance (ANOVA; *p* < 0.05) followed by Tukey’s HSD test was used to test the significance of differences in particular physiological parameters of BSC across three studied stages of succession, i.e., initial (dune ridges), middle, and late. Student’s *t*-tests (*p* < 0.05) were performed to test the significance of differences in particular physiological parameters of BSC between dune ridges and dune blowouts in the initial stage of succession. Mann–Whitney *U* test (*p* < 0.05) was used alternatively when the assumptions of the analysis were not met. Prior to the above-mentioned analyses, the distribution normality was verified with Kolmogorov–Smirnov test, and Levene’s test was performed to assess the homogeneity of variance across groups.

The relationship between chl *a* + *b* concentration (µg g^−1^ DW) and concentration of TPF (µg g^−1^ DW) were tested with Pearson’s correlation coefficients. The analysis was done on the whole data matrix including all succession stages and separately for individual stages. The data matrix for this analysis included mean values for each sampling site. Statistical analyses were performed using STATISTICA 13 (TIBCO Software Inc., USA).

## Results

### Changes in Photosynthetic Biomass and Overall Microbial Activity Along with Succession

**Fig. 4:**
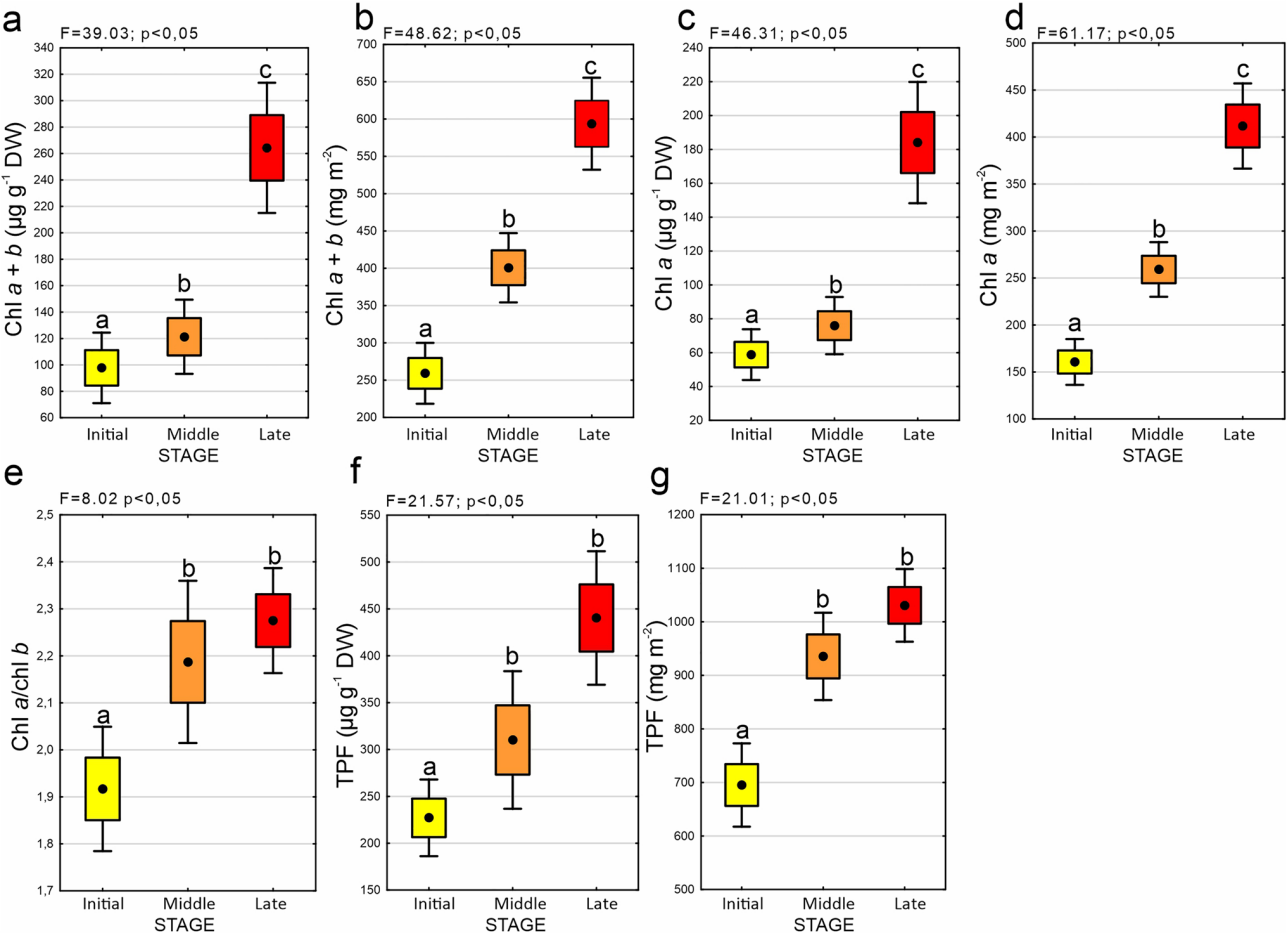
Parameters related to BSC collected from different succession stages: initial (dune ridge), middle, and late (dot = mean, box = SE, whisker = 95% confidence interval, *n* = 85): (**a**) chlorophyll *a* + *b* concentration (µg g^−1^ DW), (**b**) chlorophyll *a* + *b* concentration (mg m^−2^), (**c**) chlorophyll *a* concentration (µg g^−1^ DW), (**d**) chlorophyll *a* concentration (mg m^−2^), (**e**) chlorophyll *a/b* ratio, (**f**) dehydrogenase activity defined as triphenylformazan (TPF) concentration (µg g^−1^ DW), and (**g**) dehydrogenase activity defined as triphenylformazan (TPF) concentration (mg m.^−2^). The results of one-way ANOVA testing the significance of differences between succession stages are provided on the graphs. The various letters above the whiskers indicate statistically significant differences (*p* < 0.05)

Regarding chl *a* + *b* concentration in the initial, middle, and late succession stages, the differences between all groups were statistically significant (Fig. 4a, b, Supplementary Table S2). We observed a similar trend with regard to concentration expressed on both dry weight (µg g^−1^ DW) (Fig. 4a) and interpolated to the surface (mg m^−2^) (Fig. 4b). The chl *a* + *b* concentrations were the lowest in the initial stage, then higher in the middle stage, reaching the highest values in the late succession stage. With regard to chl *a* measured both on dry weight and interpolated to the surface, the concentrations differed significantly between the initial, middle, and late succession stages (Fig. 4c, d). In both cases, significantly higher concentrations were recorded in the late succession stage compared to the initial stage. Comparing the results on dry weight and the surface, in the case of both concentrations of chl *a* + *b* and chl *a* alone, the differences between the individual succession stages were more pronounced in concentrations interpolated to the surface, and a distinct increase with succession stage could be observed (Fig. 4b, d). Regarding the chl *a*/*b* ratio in the initial, middle, and late succession stages, the differences were statistically significant (Fig. 4e). In detail, the lowest values were observed in the initial stage, and it differed significantly from the middle and late succession stages (Fig. 4e).

As regards TPF concentrations which are proxy for dehydrogenase activity, the differences between initial, middle, and late succession stages were statistically significant (Fig. 4f, g, Supplementary Table S2). In the case of results both on dry weight and interpolated to the surface, the concentration of TPF increased with the succession stage, but the middle and late stages did not differ statistically significantly.

### Comparison of Dune Ridges and Blowouts

**Fig. 5:**
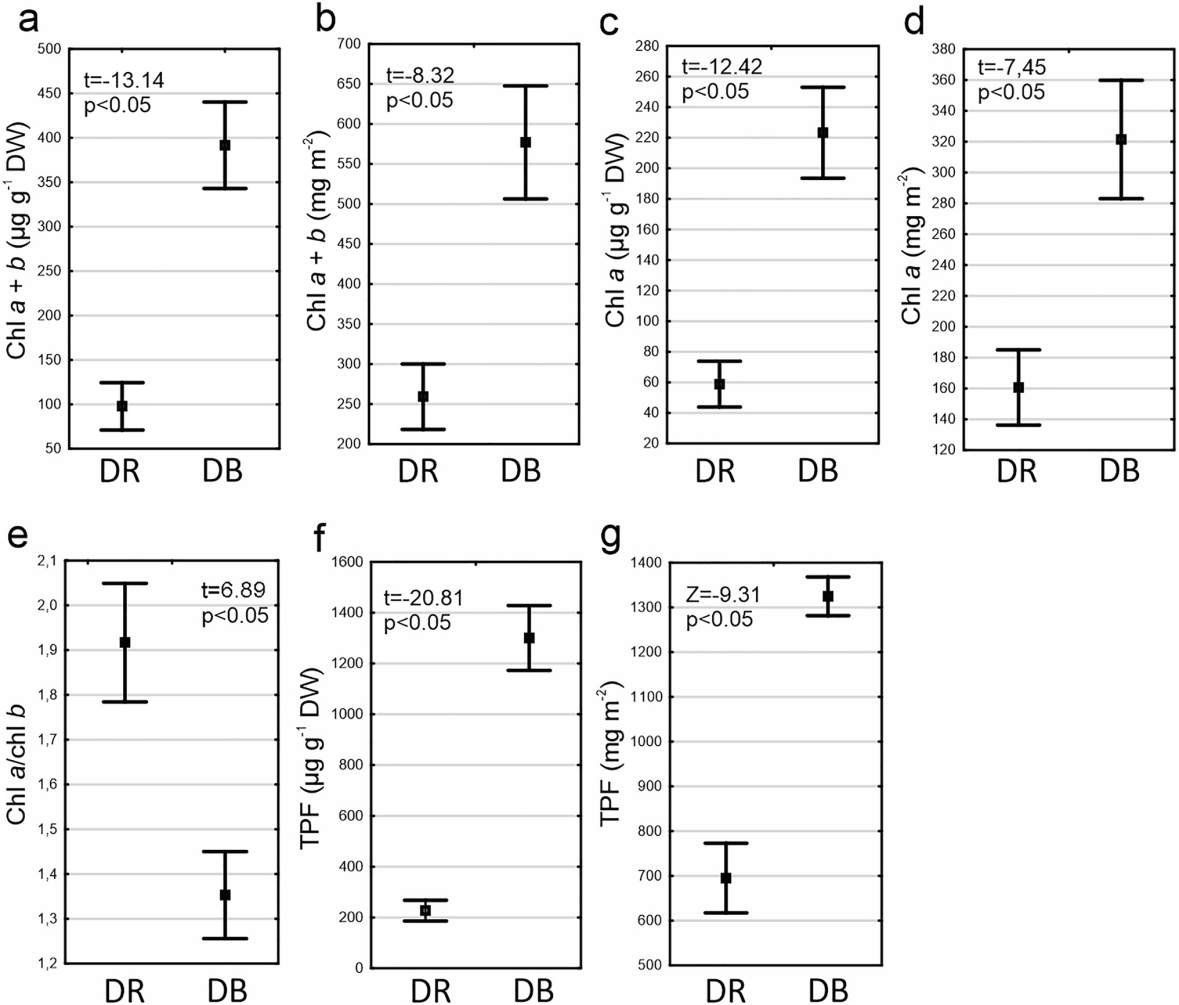
Parameters related to BSC collected from the initial succession stage at dune ridges (DR) and dune blowouts (DB) (dot = mean, box = SE, whisker = 95% confidence interval, *n* = 85): (**a**) chlorophyll *a* + *b* concentration (µg g^−1^ DW), (**b**) chlorophyll *a* + *b* concentration (mg m^−2^), (**c**) chlorophyll *a* concentration (µg g^−1^ DW), (**d**) chlorophyll *a* concentration (mg m^−2^), (**e**) chlorophyll *a/b* ratio, (**f**) dehydrogenase activity defined as triphenylformazan (TPF) concentration (µg g^−1^ DW), and (**g**) dehydrogenase activity defined as triphenylformazan (TPF) concentration (mg m^−2^). The results of Student’s *t*-tests/Mann–Whitney *U* test assessing the significance of differences between dune ridges and dune blowouts are provided on the graphs

Regarding chl *a* + *b* concentrations, significant differences between dune ridges and dune blowouts were found (Fig. 5a, b, Supplementary Table S2). Significantly higher chl *a* + *b* concentrations, measured both on dry weight and interpolated to the surface, were recorded in dune blowouts compared to dune ridges (Fig. 5a, b). Moreover, the concentration expressed on dry weight was nearly four times higher in dune blowouts than in dune ridges. A similar trend was observed in the case of chl *a* concentration (Fig. 5c, d). In this case also, differences between groups were statistically significant. With regard to the chl *a*/*b* ratio measured in BSC from dune ridges and dune blowouts, the reverse trend compared to the previous result was observed. Chl *a*/*b* ratio in dune ridges was significantly higher than observed dune blowouts (Fig. 5e).

Regarding TPF concentration which is a proxy for dehydrogenase activity, significant differences between dune ridges and dune blowouts were found (Fig. 5f, g, Supplementary Table S2). Significantly higher DHA measured both on dry weight and interpolated to the surface were recorded in dune blowouts compared to dune ridges (Fig. 5f, g).

### Relationships Between Photosynthetic Biomass and Microbial Activity

**Fig. 6:**
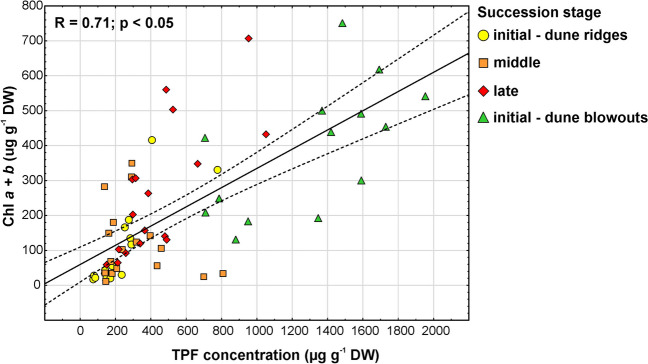
Relationship between chl *a* + *b* and TPF concentrations measured in BSC originated from different succession stages. The dotted lines indicate a 95% confidence interval. Pearson’s correlation coefficient (*R*) and *p* value are provided

As regards the relationships between chl *a* + *b* and TPF concentrations, the parameters were significantly and positively correlated (*R* = 0.71; *p* < 0.05), which indicates that DHA increased with increasing photosynthetic biomass (Fig. 6). Considering the same relationship in particular stages of succession, a significant positive correlation was observed in the case of the initial stage for dune ridges (*R* = 0.64; *p* < 0.05), and for dune blowouts (*R* = 0.46; *p* < 0.05) as well as in the late succession stage (*R* = 0.51; *p* < 0.05). Only in the middle stage, the correlation was not significant (*p* > 0.05).

## Discussion

Biological soil crusts form specific conglomerates consisting of many autotrophic and heterotrophic organisms. They are often the initial colonizers of developing or degraded terrestrial environments as they can withstand the unfavorable habitat conditions typical of early succession stages [4]. The emergence of BSC begins to change the ecosystem, and as BSCs develop, various organisms enter into dynamic and complex interactions, resulting in changes in species composition and biodiversity of the macro- (bryophyte and lichens) and microorganisms (algae, bacteria, and fungi) along with succession [41]. Chlorophyll is a pigment commonly occurring in autotrophic organisms which facilitates organisms to utilize sunlight as an energy source to build carbohydrates from CO_2_ and water. Chlorophyll concentrations in BSC are commonly used to quantify the relevance of photosynthetically active organisms within these communities [5]. The chlorophyll content of these communities is therefore a good indicator of the photosynthetic capacity and the capability of these systems to acquire energy [40]. Chlorophyll *a* content in BSC was found to be significantly higher in crusted soils compared to bare soil [42]. With the development and succession of BSC, photoautotrophic biomass also varies. It was also found that chl *a* and chl *a* + *b* considerably increase from an early successional stage [43]. Contrary, no significant differences in chlorophyll concentration between initial and stable BSCs were observed by Langhans et al. [8] in temperate sand ecosystems. Our results revealed a significant increase of both chl *a* + *b* and chl *a* as the succession of inland shifting sands progressed. The chl *a* content of BSCs is often used as a criterion for measuring the total density of crust organisms. It varies from under 100 up to 900 mg m^−2^, depending on BSC species composition [5]. The concentration of chl *a* in our study ranged from 20 to 1124 mg m^−2^. This is a much wider range; however, the highest chl *a* content was recorded by us within specific dune blowout microhabitats. Our study also revealed a significant positive correlation between photosynthetic biomass and overall microbial activity. This indicates that the increase in photosynthetic biomass is closely related to increased microbial activity in the BSCs and suggests that autotrophs constitute a major BSC component. BSC is composed of both photoautotrophic cyanobacteria, algae, lichens, and bryophytes and heterotrophic fungi, bacteria, and archaea [1]. With the formation of BSCs and the increase of photoautotrophic organisms, organic matter gradually accumulates in BSC, which also accelerates the growth of heterotrophic microorganisms. In turn, heterotrophs transform organic matter into inorganic compounds, providing a more favorable condition for photoautotrophic organisms, thus forming a positive feedback mechanism [44]. Consequently, chl *a* concentration could be considered a useful quantitative indicator of both the presence of photoautotrophs and the degree of soil crust development in warm-summer humid continental climates.

We hypothesized that the chl *a*/*b* ratio would be clearly higher in the initial stages of sand colonization compared to later successional stages. We based our assumption on the fact that cyanobacteria only comprise chlorophyll *a* and are usually reported as dominant in the initial stages of succession. Thus, we suspected that a large contribution of blue-green algae would cause disturbed proportions of chl *a/b*, and there would be much more chl *a* in relation to chl *b*, and consequently, this ratio would reach higher values compared to BSCs where lichens and bryophytes dominated. Our results unexpectedly showed that it is exactly the opposite, and the lowest chl *a/b* ratio values were recorded in the initial stages of succession. This may suggest that the relevance of eukaryotic organisms containing chl *b* in the initial stages of succession is relatively high. This could be supported by the results of Rahmonov [35] and Cabała and Rahmonov [45], who found a large abundance of filamentous green algae *Cylindrocapsa* sp. forming characteristic cotton-like clusters directly on the sand surface in the Błędowska Desert. Moreover, they considered representatives of this genus to be the most important species in the succession process in its initial stages [35]. The trend of increasing chl *a/b* ratio with succession that we observed may not be true for BSC in other climatic conditions. For example, in the semi-arid climate in the Colorado Plateau (USA), mature dark crusts from the late succession stage contained greater chl *a* concentration owing to high cyanobacterial biomass and darker pigmented cyanobacterial species compared to early succession poorly developed light crusts [21]. Moreover, it is also worth mentioning that the chl *a/b* ratio in algae, lichens with green algae as photosynthetic partner, and bryophytes is also highly variable [46], and therefore, we could conclude that the chl *a/b* ratio cannot be considered a good indicator of BSC developmental stage.

Dehydrogenase is the major representative of the oxidoreductase enzyme class [47] and constitutes an intracellular enzyme present only in active cells. They indicate overall soil microbial activity and are proportional to the biomass of the microorganisms in the soil [20]. Determination of dehydrogenase activity (DHA) gives us a large amount of information about the biological characteristics of the substrate as it is associated with the abundance and metabolic activity of microorganisms. DHA also reflects the capacity to transport electrons generated in intracellular metabolic processes [22]. Most studies concerned the effect of BSC on DHA in soil, and a number of studies have shown that DHA in soil harvested from under BSC is higher (e.g., [23, 24]). However, there are only a few studies analyzing DHA in BSC samples. Previous studies that analyzed DHA in the crusts and in the soil underlying BSC showed that DHA was mostly concentrated within the BSC thickness in contrast to low enzymatic activity in the bare soil [22, 23]. Our study showed that DHA increased significantly as succession progressed. The same trend was observed in a semi-arid thermo-Mediterranean climate, where enzyme activities in BSC were close to zero in the bare substrate and reached the highest values in the final development stage of the crust [48]. The increase of DHA with the succession process is certainly associated with the increase of organic matter, which promotes greater microbial biomass since organic matter provides energy for microbial growth and enzyme production [49]. Moreover, as succession starts, the initial state constitutes bare and barren sand, which is colonized by cyanobacteria and algae, and in such consolidated crusts, the share of sand in relation to organic matter is significant [35]. This could explain the greatest increase of DHA observed in our study between the initial and middle stages of succession.

The results of our study showed that various microhabitats of inland sand ecosystems, where aeolian processes are active, differ significantly in terms of photosynthetic biomass and microbial activity. Although both dune ridges and blowouts represent initial succession stages, heterogeneity of geomorphological forms of sand dunes and associated microclimatic conditions results in mosaic differentiation of BSC physiological activity. In the initial stages of succession, where aeolian processes are still active, numerous deflation forms and dune hillocks are formed. Our study showed that BSCs formed in dune blowouts are characterized by 4 times higher photosynthetic biomass and 6 times higher overall microbial activity than those formed on dune ridges. The blowout is a saucer cup- or trough-shaped hollow formed by wind erosion on a preexisting sand deposit [50]. This results in different microhabitat conditions for BSC development. The high biomass of microorganisms in the dune blowouts may be associated with a high amount of allochthonous organic material mainly brought into the blowout by rain splash or surface runoff and more favorable moisture conditions. This is consistent with the observations of Kammann et al. [51] who found that the increase in the organic matter and moisture content leads to advanced biocrust development. Soil moisture plays an important role because even small amounts of water can promote the stimulation of microorganisms. Miralles et al. [52] found that soil moisture promotes enzymatic activities in BSC and revealed a significant and positive correlation between soil moisture and DHA in BSC. High moisture content in soil could also increase soil-soluble organic matter, which may be responsible for the increase in bacterial populations [53]. Since dune blowouts constitute concave geomorphological forms, rainwater flows down the slopes of the dunes and accumulates at least temporarily in dune blowouts, providing the BSC with more favorable conditions for microorganisms to develop. Moreover, a higher amount of organic matter accumulated in dune blowouts could also accelerate the growth of heterotrophic microbes. Enzymatic activity is strongly related to the organic matter content in BSC. High organic matter content promotes greater microbial biomass and thereby influences DHA [54, 55], which could explain the very high DHA in BSC developed in dune blowouts. All these make dune blowouts environmental niches facilitating the development of microorganisms forming BSC, compared to dune ridges. The high porosity of the sands leads to the rapid infiltration of rainwater, preventing water from accumulating on the surface, which limits access to this water source for photoautotrophs forming BSC. Although BSC organisms have extraordinary abilities to survive desiccation, UV radiation, and extreme temperatures [56, 57], limited availability of water slows down the development of BSC and could even stop the succession at a certain state [58].

## Conclusions

Our study revealed a significant increase in both photosynthetic biomass and overall microbial activity in BSC as the succession of inland shifting sands progresses. We can conclude that chl *a* concentration in BSC could be considered a useful quantitative indicator of both the presence of photoautotrophs and the degree of soil crust development in warm-summer humid continental climates. We found that photosynthetic biomass is closely related to increased microbial activity in the BSCs, which suggests that autotrophs constitute a major BSC component. Unexpectedly, the lowest chl *a/b* ratio was recorded in the initial stages of succession, which indicates the relevance of eukaryotic organisms containing chl *b* in the initial stages of sand colonization. Although both dune ridges and blowouts represent initial succession stages, heterogeneity of geomorphological forms of sand dunes and associated microclimatic conditions results in mosaic differentiation of BSC physiological activity. Dune blowouts constitute environmental niches facilitating the development of microorganisms forming BSC, compared to dune ridges in which organisms experience exposure to more extreme conditions. A high biomass of microorganisms in the dune blowouts may be associated with a high amount of allochthonous organic material and more favorable moisture conditions. We conclude that deflation fields are key places for keeping a mosaic of habitats in the area of shifting sands and can be a reservoir of microorganisms supporting further settlement of dune slopes by BSC.

### Supplementary Information

Below is the link to the electronic supplementary material.

Supplementary file1 (PDF 170 KB)

## Data Availability

The datasets generated during and/or analyzed during the current study are available from the corresponding author upon reasonable request.
