# Supplementary material for: Dune Blowouts as Microbial Hotspots and the Changes of Overall Microbial Activity and Photosynthetic Biomass Along with Succession of Biological Soil Crusts
Source: Microb Ecol. 2023 Dec 29;87(1):22. doi: 10.1007/s00248-023-02333-4 (PMC10756888; doi:10.1007/s00248-023-02333-4)
Supplement: Supplementary file 1 — Supplementary file1 (PDF 170 KB) [file 248_2023_2333_MOESM1_ESM.pdf]

SUPPLEMENTARY MATERIALS TO THE ARTICLE

Dune blowouts as microbial hotspots and the changes of overall microbial activity and photosynthetic biomass along with succession of biological soil crusts

Karolina Chowaniec[1,2], Jakub Styburski[1,2], Szymon Kozioł[1], Zofia Pisańska[1], Kaja Skubała[1]*

[1]Institute of Botany, Faculty of Biology, Jagiellonian University, Gronostajowa 3, 30-387 Kraków, Poland
[2]Doctoral School of Exact and Natural Sciences, Jagiellonian University in Kraków, prof. S. Łojasiewicza 11, 30-348 Kraków, Poland
*Corresponding author. E-mail address: kaja.skubala@uj.edu.pl (K. Skubała)

Table S1. The selected climatic parameters measured at two different meteorological stations near the study area (IMGW codes: 250190390 and 350190566) averaged for 10-year periods (means ± SE). Raw data source: The Institute of Meteorology and Water Management - National Research Institute 2023.

Decade	Average monthly temperature [°C]	Monthly rainfall [mm]	Number of days with rainfall	Average monthly relative humidity [%]	Average monthly wind speed [m/s]	Number of days with wind speed ≥10 m/s*	Number of days with wind speed ≥15 m/s*	Number of days with dew*
2022-2013	10.24±0.06	60.77±0.36	10.72±0.04	74.87±0.07	1.753±0.003	3.57±0.03	0.142±0.003	10.18±0.07
2012-2003	9.33±0.07	57.56±0.36	10.64±0.05	75.64±0.07	1.619±0.003	2.90±0.02	0.142±0.004	11.12±0.08

2002-1993	9.00±0.06	58.51±0.36	10.72±0.05	76.80±0.06	1.555±0.003	2.94±0.03	0.133±0.003	13.53±0.08
1992-1983	8.72±0.06	51.23±0.30	10.18±0.04	77.85±0.04	1.516±0.003	1.20±0.01	0.042±0.002	14.17±0.09
1982-1973	8.35±0.06	53.08±0.26	10.32±0.05	78.75±0.05	1.822±0.004	0.71±0.01	0.017±0.001	13.58±0.09
1972-1963	8.19±0.07	64.14±0.35	10.35±0.05	79.96±0.05	1.927±0.004	N/A	N/A	N/A
1962-1953	8.44±0.06	56.13±0.34	10.04±0.04	80.18±0.08	1.683±0.005	N/A	N/A	N/A
**Mean value for given period (2022-1953 or 2022-1973)**	**8.90±0.01**	**57.36±0.05**	**10.42±0.01**	**77.72±0.01**	**1.696±0.001**	**2.263±0.004**	**0.095±0.001**	**12.51±0.02**

*Due to lack of data for the period 1953-1965 no data were provided.
N/A - not available

Table S2. The parameters related to photosynthetic biomass and overall microbial activity of BSC collected from different succession stages (mean ± SE, n = 85, minimum and maximum values).

Succession stage		Initial		Middle	Late
Parameter		Dune ridge	Dune blowout		
Chlorophyll a + b (µg g-1 DW)	Mean±SE	97.75±13.42	391.63±24.4	121.29±14.1	264.29±24.771
	Min-Max	8.99-498.85	101.03-968.79	8.88-734.5	30.48-949.12
Chlorophyll *a* + *b* (mg m^{-2})	Mean±SE	259.16±20.54	576.96±35.4	400.68±23.35	593.72±30.99
	Min-Max	29.97-842.77	100.09-1511.56	25.26-883.99	201.82-1538.05
Chlorophyl *a* (µg g^{-1} DW)	Mean±SE	58.84±7.54	223.28±14.91	75.97±8.5	184.04±17,01
	Min-Max	6.28-258.48	49.25-627.28	6.74-469.08	22.47-684.07
Chlorophyl *a* (mg m^{-2})	Mean±SE	160.62±12.26	321.42±19.25	259.07±14.61	411.76±22,8
	Min-Max	21.39-490.45	48-771.32	20.44-625.42	136.87-1123.48
Chlorophyl *a/b* ratio	Mean±SE	1.92±0.07	1.35±0.05	2.19±0.09	2.28±0.06
	Min-Max	0.84-3.20	0.77-2.48	0.99-4.53	0.97-3.52
Dehydrogenase activity - triphenylformazan (µg g^{-1} DW)	Mean±SE	227.07±20.56	1300.35±64	310.15±36.92	440.29±35.81
	Min-Max	39.54-20.56	515.08-2729.53	35.54-2550.49	100.09-1742.8
Dehydrogenase activity - triphenylformazan (mg m^{-2})	Mean±SE	695.2±39.1	1342.74±21.67	935.36±441	1030.71±34.12
	Min-Max	123.75-1509.73	495.77-1526.01	209.56-1527.26	317.28-1544.80
